# Phosphoprotein analysis: from proteins to proteomes

**DOI:** 10.1186/1477-5956-4-15

**Published:** 2006-07-19

**Authors:** Frédéric Delom, Eric Chevet

**Affiliations:** 1 Dept of Surgery, McGill University, Montreal, Quebec, Canada; 2 Montreal Proteomics Network, McGill University, Montreal, Quebec, Canada; 3 Dept of Medicine, McGill University, Montreal, Quebec, Canada; 4 Dept of Anatomy, McGill University, Montreal, Quebec, Canada

## Abstract

Characterization of protein modification by phosphorylation is one of the major tasks that have to be accomplished in the post-genomic era. Phosphorylation is a key reversible modification occurring mainly on serine, threonine and tyrosine residues that can regulate enzymatic activity, subcellular localization, complex formation and degradation of proteins. The understanding of the regulatory role played by phosphorylation begins with the discovery and identification of phosphoproteins and then by determining how, where and when these phosphorylation events take place. Because phosphorylation is a dynamic process difficult to quantify, we must at first acquire an inventory of phosphoproteins and characterize their phosphorylation sites. Several experimental strategies can be used to explore the phosphorylation status of proteins from individual moieties to phosphoproteomes. In this review, we will examine and catalogue how proteomics techniques can be used to answer specific questions related to protein phosphorylation. Hence, we will discuss the different methods for enrichment of phospho-proteins and -peptides, and then the various technologies for their identification, quantitation and validation.

## Background

Post-translational modifications of proteins are considered to be one of the major determinants regarding organisms complexity [1]. To date, at least more than 200 different types of post translational modifications (PTM) have been identified of which only a few are reversible and important for the regulation of biological processes. One of the most studied PTM is protein phosphorylation, because it is vital for a large number of protein functions that are important to cellular processes spanning from signal transduction, cell differentiation, and development to cell cycle control and metabolism. A primary role of phosphorylation is to act as a switch to turn "on" or "off" a protein activity or a cellular pathway in an acute and reversible manner [2]. Furthermore, it is estimated that one of every three proteins is phosphorylated at some point in its life cycle [3]. Today, it is well-known that almost all processes regulated by protein phosphorylation are reversible and controlled by the combined action of two different classes of enzymes, namely protein kinases and phosphatases. These kinases and phosphatases, constitute about 2% of the human genome [1,4,5].

Analysis of the entire cellular phosphoproteins panel, the so-called phosphoproteome, has been an attractive study subject since the discovery of phosphorylation as a key regulatory mechanism of cell life. But despite a growing knowledge of many phosphorylation consensus sequences, this PTM cannot usually be predicted accurately from the translated gene sequence alone. Thus, the experimental determination of phosphorylation sites is an important task. To this end, the development and optimization of protocols for the enrichment of phosphorylated proteins or peptides is essential. In addition, various methods for protein phosphorylation site determination have been developed, yet this task remains a technical challenge [6]. Well established methods involving the analysis of ^32^P-labeled phosphoproteins by Edman degradation and two-dimensional phosphopeptide mapping have proven to be powerful but not without limitations. Beyond the inconvenience associated to the use of radioactivity, these traditional phosphorylation analysis methods can be time-consuming and are not well suited for the high throughput pipelines required for phosphoproteome analysis. Consequently, mass spectrometry has emerged as a reliable and sensitive method for the characterization of protein phosphorylation sites [7] and may therefore represent a method of choice for the analysis of protein phosphorylation [8].

Unfortunately, phosphoproteins analysis is not straightforward for five main reasons. First, the stoichiometry of phosphorylation is generally relatively low, because only a small fraction of the available intracellular pool of a protein is phosphorylated at any given time as a result of a stimulus. Second, the phosphorylatation sites on proteins might vary, implying that any given phosphoprotein is heterogeneous (i.e. it exists in several different phosphorylated forms). Third, many of the signaling molecules, which are major targets of phosphorylation events [9], are present at low abundance within cells and, in these cases; enrichment is a prerequisite before analysis. Fourth, most analytical techniques used for studying protein phosphorylation have a limited dynamic range, which means that although major phosphorylation sites might be located easily, minor sites might be difficult to identify. Finally, phosphatases could dephosphorylate residues unless precautions are taken to inhibit their activity during preparation and purification steps of cell lysates.

In this review, we present at first a survey of methods available to identify phosphoproteins and phosphopeptides and to map the precise phosphorylated residues and secondly, we enumerate methodologies available to quantitate and validate the identified phosphorylation sites/events.

## Review

**Figure 1:**
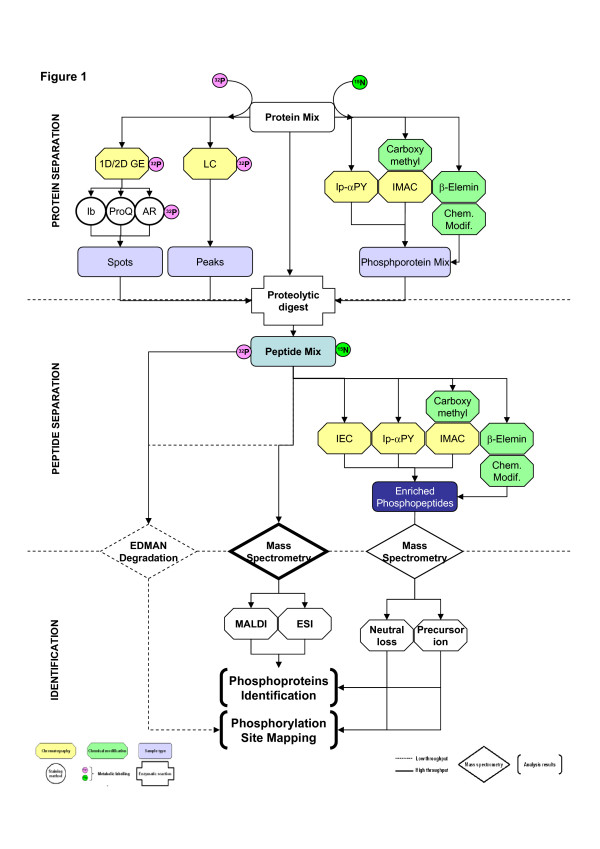
**Schematic representation of phosphoprotein analysis workflow**. The most commonly used methods to resolve, to purify or to enrich phosphoproteins are described in the **Protein separation section**. Peptides resulting from trypsin digestion of phosphoproteins may be analyzed directly by MS or by Edman degradation. Other approaches may be used for the enrichment of phosphopeptides. These methods are depicted in the **Peptide separation section**. MALDI and ESI are usually used for identification of phosphoproteins. The two predominant techniques for idenditification of the precise sites of phosphorylation are i) Edman degradation and ii) MS analysis (Neutral loss and Precursor ion). These methods are described in the **Identification section**. **1D/2D GE**: On-dimensional/Two-dimensional gel electrophoresis. **Chem. Modif**.: Chemical Modification. **ESI**: Electrospray Ionisation. **Ib**: Immunoblot. **IMAC**: Immobilized Metal Affinity Chromatography. **Ip-α PY**: Immunopurification using a phospho-tyrosine antibody. **LC**: Liquid Chromatography. **MALDI**: Matrix-Assisted Laser Desorption Ionization. **ProQ**: Pro-Q Diamond™. **AR**: Autoradiography. **β-Elemin**: β-elimination reaction.

The identification of phosphoproteins and the characterization of their phosphorylation sites was greatly improved by the introduction of mass spectrometry, but only a fraction of the proteins in a proteome are phosphorylated at any given time. Some of the most commonly used methods for enrichment of phosphoproteins or phosphopeptides, when limiting amounts are available, will now be discussed. These steps can be coupled to various analytical methods for detection and micro-characterization. An overview of the experimental workflow described below is presented in Figure 1.

## Phosphoprotein separation and detection

### Two-dimensional gel electrophoresis (2-DGE)

Normally, for separation by 2-DGE, proteins are subjected to isoelectric focusing and separated by size. Using this approach, a cell extract is prepared from two different samples and resolved by 2-DGE for comparison of protein expression or changes in protein modification by comparison of the two corresponding gels. Currently, 2-D PAGE is the only method capable of simultaneously resolving several thousands of proteins [10,11] including protein variants produced by the co- or post-translational processing such as phosphorylation, glycosylation, and sulfation [12]. The phosphorylation of a protein leads to a decrease in its pI and consequently its coordinates in a 2-D gel. To map phosphoproteins on 2-DGE, it has been exploited this fact to discriminate phosphoproteins from nonphosphoproteins. Although this method can provide valuable information, it suffers from many limitations, including poor protein representation and an inability to identify low-copy proteins [13,14]. Another limitation to 2-DGE resides in the fact that only some proteins with molecular weight between 10 and 100 kDa are visualized. Furthermore, 2-DGE is poorly suitable to resolve integral membrane proteins due to proteins aggregation during the first isoelectric-focusing (IEF) migration. Thus, the limitations of 2-DGE have inspired the development of several methods (see below).

### Immunoblot

Immuno- or Western blot is a technique [15] which requires the availability of specific antibodies to detected proteins transferred from a one-dimensional gel electrophoresis (1-DGE) or 2-DGE [16] to a solid membrane support. The development of antibodies against common protein epitopes allows the identification of proteins sharing the same characteristics such as phosphorylated proteins. For phosphoproteome analysis phosphoserine, phosphothreonine and phosphotyrosine represent the common epitopes which are recognized by specific antibodies that are routinely employed. Analysis of phosphoproteome using Western blot is improved by combining 2-DGE with highly selective anti-phosphoantibodies [17]. While excellent anti-phosphotyrosine antibodies are available, better anti-phosphoserine and anti-phosphothreonine antibodies are currently needed. This is probably one of the major reasons why tyrosine phosphorylation, which is much less frequent in cells than serine/threonine phosphorylation, is much more studied [18-20]. Although Western blot allows the detection of very low abundance phosphoproteins, this method is not very suitable for quantitative analysis due to the variability of the amount of proteins transferred to the membrane. In addition, the selectivity and affinity characteristics of the antibodies are of major importance since a large number of "false positive" interactions may be detected, thus reducing the applicability of this approach.

### Direct staining

The apparently easiest way to analyze phosphoproteome of cells, tissues or organisms is to employ reagents designed to selectively detect phosphoproteins directly in 1- or 2-DGE. Since 1970 several methods have been described [21,22] to stain phosphoproteins directly in gels but their low specificity and sensitivity prevented those methods from being routinely applied. Recently, a fluorescent phosphosensor dye, Pro-Q Diamond™ has been developed [23]. This dye can discriminate between phosphorylated and unphosphorylated proteins and is reversible. In addition, it is compatible with protein dyes such as SYPRO Ruby. Pro-Q Diamond™ combined to SYPRO Ruby dye allows first, to detect phosphorylated proteins (Pro-Q Diamond™) and total proteins (SYPRO Ruby) on the same gel and second, to distinguish between a slightly phosphorylated from high abundant protein and a highly phosphorylated from a low abundant protein by comparing the results of the two different colorations[24,25]. This dye permits phosphoproteins identification in a complex protein mixture with sensitivity in the ng/ml range. Although this sensitivity is significantly good, it is however not sufficient for comprehensive analysis of the phosphoproteome.

### Protein phosphatases

Phosphorylation of proteins leads to a change of the net charge of proteins and thus the migration behavior during 2-DGE. Accordingly, the charge variation occurring after phosphatase treatment can be exploited to discriminate phosphorylated from unphosphorylated proteins. Phosphatase treated and untreated samples are analyzed by 2-DGE and the resulting 2D maps compared in order to detect differences in migration corresponding to phosphorylated proteins. This experimental strategy capitalizes on the specific enzymatic activity of k-phosphatase (kPPase) on phosphoserine, phosphothreonine, phosphotyrosine and phosphohistidine residues [26]. An improvement of this experimental strategy was recently achieved by employing difference gel electrophoresis (DIGE) technology for the detection of variations in protein migration after kPPase treatment [27]. Since DIGE eliminates gel-to-gel variability thus allowing separation of two proteomes on the same gel, detection of changes in protein patterns is greatly facilitated. The phosphatase-based method allows an easy identification of phosphorylated proteins expressed in a sample but it is less suitable for the quantification of variations of protein phosphorylation patterns comparing two different samples. The complexity of the analysis and the variability of the efficacy of enzymatic action are the main reasons for this.

### Isotopic labeling

Protein labeling with either inorganic phosphate (^32^P_i_) *in vivo *or γ-(^32^P)-ATP *in vitro *is still one of the most practical way to study protein phosphorylation [28-33]. Separation of labeled proteins by 1- or 2-DGE and autoradiography or image acquisition by PhosphorImager systems for visualization of phosphorylated proteins is the most common workflow pursued in proteomics studies when an isotopic labeling strategy is considered.

#### Analysis of protein phosphorylation in vivo

In this approach, cells are labeled *in vivo *with ^32^P-orthophosphate in pulse or pulse-chase experiments in cell/tissue culture [34,35]. Then, cell extracts are prepared and analyzed. The *in vivo *phosphorylation, while more physiologically relevant by avoiding the possibility of non-specific phosphorylation, requires working with significantly larger amounts of radioactivity due to the extensive cellular consumption of phosphate in the form of phosphoproteins, nucleic acids, phospholipids, etc. However, *in vivo *radiolabeling has limitations: (i) ^32^P-labeling is inefficient because of the high concentrations of non-radioactive endogenous ATP; (ii) although many phosphoproteins can be visualized by autoradiography, they are not present in sufficient amounts to be identified, and (iii) the use of a cell/tissue culture.

#### Protein kinase profiling in vitro

Protein kinase profiling is a way to identify protein kinases and of course, their substrates under various biological conditions. In this strategy, radiolabeled phosphoproteins are generated *in vitro*, using γ-^32^P-ATP and either purified protein kinase or cell lysates, and then are analyzed [36,37]. The advantage of studying *in vitro *phosphorylation is the theoretical goal 100% phosphorylation stoichiometry. However, *in vitro *phosphorylation has limitations in that phosphorylation sites may differ from what might take place *in vivo*. Thus, the big disadvantage is the burden of proof that the phosphorylation sites found are real and relevant physiologically. As a consequence, the results must, in general, be confirmed by *in vivo *studies.

## Phosphoproteome enrichment and identification

The limitations of gel electrophoresis have inspired the development of methods to circumvent protein gels entirely. In one such method, the entire protein mixture isolated from cells is converted to peptides, which are then resolved by liquid chromatography. Following separation, the peptides are injected directly into a mass spectrometer in an "on-line" configuration for mass analysis and protein identification. A variety of techniques have emerged using this general strategy, including multidimensional liquid chromatography [38], cation-exchange and reverse-phase chromatography [39], and liquid chromatography combined with ion mobility spectrometry and tandem mass spectrometry (LC-IMS-TOF-MS/MS) [40]. The advantage of these methods is that, theoretically, more proteins can be identified, especially low-copy proteins. However, protein gels perform an important function in that they allow for the visual selection of specific proteins from a complex mixture.

### Methods for phosphoproteome enrichment

A major obstacle in the study of phosphorylated proteins is that they comprise only a small fraction of the total protein in a cellular lysate. Thus, many phosphoproteins cannot be identified in a cell extract. As a consequence, a number of techniques have been developed to partially purify or to preferentially enrich phosphopeptides from a complexe mixture, namely i) Immobilized Metal Affinity Chromatography (IMAC); ii) Specific chemical derivatization; and iii) Immunoprecipitation.

#### Immobilized Metal Affinity Chromatography (IMAC)

The use of (miniaturized) immobilized metal affinity chromatography (IMAC) columns was developed for the enrichment of phosphopeptides and exploits the high affinity of a phosphate group to cations such as Zn^2+^, Fe^3+^, and Ga^3+ ^[41,42]. IMAC has been successfully used either in off-line or on-line formats for the detection of phosphopeptides using MS [42-45]. Although this approach is useful, it does have a problem in that it is not absolutely specific because peptides that contain many acidic amino-acids, histidine or cysteine co-elute [46]. In addition, multiply phosphorylated peptides are more enriched and the recovery of phosphopeptides appears to be largely dependent on the type of metal ion, column material and the elution procedure used. Other IMAC approach has permitted a much higher specificity using esterification of acidic residues before IMAC enrichment [47]. This way eliminated the non-specific binding, and enabled the determination of a large number of phosphorylation sites on proteins from whole-cell lysates. Moreover, this approach requires that all phosphoproteins in a cell lysate have to be analyzed to detect those that undergo changes. Recently, Larsen and colleagues reported the use of Titanium dioxide (TiO_2_) as a potent chelator for phosphopeptides which may be used upstream of mass spectrometry sequencing [48]. In this work, the authors attributed the selective enhancement of phosphorylated peptide binding by dihydroxybenzoic acid to an effective competition predominantly with non-phosphorylated peptides for binding sites on TiO_2_. This effect was thought to be achieved by the existence of a heterogeneous array of adsorption sites on TiO_2_. In direct comparison with IMAC, this procedure proved superior in terms of selectivity and sensitivity of phosphorylated peptide binding [48]. In addition, the TiO_2 _purification was fast and can be used in combination with high performance liquid chromatography coupled to either MALDI-MSMS or ESI-MSMS.

#### Specific chemical derivatization

One approach to isolate phosphorylated proteins or peptides is to take advantage of the unique chemistry of phosphoamino acids in peptides. To date, two methods have been reported that use chemical modification of the phosphate moiety as a strategy to enrich phosphopeptides from complex mixtures. The first method uses a β-elimination reaction that occurs when phosphoserine and phosphothreonine residues are exposed to strong alkaline conditions [49,50]. The resulting dehydroalanine or dehydroaminobutyric acid residues can be detected, after chemical modification with ethanethiol (EDT), using tandem mass spectrometry (MS/MS) [51,52]. The same strategy can be used to attach biotinylated moieties to purify phosphoproteins or peptides. Thus, EDT is used as a nucleophile, which provides a new reactive thiol group serving as a linker for attachment of a biotinylated affinity tag [49]. However, an undesired side effect involving side chains on cysteine and methionine residues can still occur. To overcome this problem, the sample is first treated with performic acid, leading to oxidation of these residues, thereby inactivating them. The second method is an alternate strategy to isolate phosphotyrosine-containing peptides in addition to those containing phosphoserine and phosphothreonine residues [53]. The main feature of this method is that a transient carbodiimide [ethyl carbodiimide (EDC)] catalyzes the addition of cystamine to phosphate moieties, which then allows purification of phosphopeptides on glass beads containing immobilized iodoacetyl groups. Elution of phosphopeptides is performed by cleavage of phosphoroamidate bonds by trifluoroacetic acid. The main disadvantage of these two methods is that the current chemistries require significant amounts of protein or peptide for identification by MS to be successful. In addition, the selectivity of these methods has not been confirmed yet. Several proteins were isolated using these methods that are not known phosphoproteins. Nevertheless, these approaches are promising and could be coupled to other fractionation steps to improve the overall recovery of low-abundance proteins.

#### Immunoprecipitation

Antibodies are routinely used to immunoprecipitate specific proteins. Consequently, phospho-specific antibodies can be used to selectively immunoprecipitate phosphorylated proteins depending on the specificity of the antibody. As for Western blot (see above) anti-phosphotyrosine antibodies are the most reliably and widely used in order to enrich tyrosine-phosphorylated proteins from complex mixtures. These antibodies can be used to immunoprecipitate, and therefore to enrich, tyrosine phosphorylated proteins from complex mixtures of proteins such as cell lysates. Although these antibodies have been relatively effective at enriching and identifying low-abundance tyrosine phosphorylated proteins [54,55], it has been showed that the existing immunopurification protocols for phospho-tyrosine (pY) containing peptides have a poor selectivity [19]. Recently, a method using a pY antibody for pY peptide purification from an enzymatically digested protein extract combined with LC-MS/MS was applied to large scale pY analysis in cancer cells [20]. Currently, there are no antibodies that are suitable for enriching proteins that are phosphorylated on serine or threonine residues, and thus these proteins must be enriched using the alternative methods described above.

### Methods for phosphoproteome identification

The use of mass spectrometry (MS) in protein phosphorylation site determination has increased significantly in the past few years and is now the dominant technology for protein identification. However, the success of protein identification depends on both the sample preparation and the type of mass spectrometer used. The two most common methods used for mass spectrometry are the matrix-assisted laser desorption ionization (MALDI) and the electrospray ionization (ESI) or the combination of both. It is evident that in most cases multiple techniques used in combination have been necessary for phosphoproteins analysis and that no single combination of approaches appears to be optimal for all proteins.

#### Mass spectrometry (MS)

Phosphorylation analysis by mass spectrometry is generally accomplished by a two-step approach. The phosphoprotein of interest is proteolytically digested, usually with trypsin, and the tryptic peptides are analyzed to determine which are phosphorylated. Then those phosphopeptides are further analyzed, usually by tandem mass spectrometry (MS/MS), to determine the precise location of the phosphorylation site(s). Phosphopeptides may be identified simply by examination of the list of observed peptide masses for mass increases of 80 Da (the added mass of the phosphate group) compared with the list of expected peptide masses. Although this method is relatively straightforward, it also misses many phosphorylated peptides because: i) The peptide maps are frequently incomplete even for non-phosphorylated proteins; ii) The increased acidity of the phosphate group generally results in decreased ionization efficiency of a peptide [56]; and iii) The competition for ionization of peptides in a mixture results in suppression of signal for some peptides. Nonetheless the ease and small amount of sample required for a simple peptide map make this method popular. Using β-elimination/Michael addition chemistry to replace the phosphate with a chemical group more conducive to efficient ionization can ameliorate some of the difficulties associated with phosphopeptides [57].

Phosphopeptide mass measurement may be achieved by MALDI, ESI or the combination of both. These processes are the two most common ways to ionize peptides, yet they differ fundamentally. For this reason, one may often find some proteins ionize better by one process than the other.

One approach to reduce the sample required for ESI measurement is to reduce the fluid flow by use of small capillary electrospray emitter tips, a process known as nanoelectrospray. Nanoelectrospray produces a constant signal for 10–30 min for a 1 μl sample, and the low flow has been shown to increase the ionization efficiency and reduce ion suppression [58]. Another way to reduce ion suppression phenomena is to separate the peptides prior to ionization. The common method is LC-MS, which has the added benefit of concentrating dilute samples and removing salt that interferes with the ionization process. As a way to highlight the presence of phosphopeptides in a mixture or to confirm the identity of a phosphopeptide, a simple phosphatase reaction will cause a downward shift in mass of 80 Da (or multiples of 80) for each phosphopeptide [59].

#### Database searching

Using data produced by mass spectrometers, proteins can be identified by searching DNA and protein-sequence databases. The success of protein identification depends on the type of data utilized, the type of search conducted, and the databases searched: i) Peptide Mass Fingerprint Database Searching is used for peptide masses obtained from MALDI-MS, which are compared against theoretical spectra obtained from primary-sequence databases; ii) Peptide mass tag database searching can be conducted using peptide sequence obtained from MS/MS. In this approach, a partial amino acid sequence, known as the sequence tag, is combined with the mass of the peptide to search relevant databases [60] and iii) the FASTS program uses amino acid sequence obtained from MS/MS to search databases from organisms whose genomes have not been sequenced completely or whose databases are not fully annotated [61].

## Phosphorylation site mapping

In addition to identifying phosphoproteins, it is important to characterize/map their phosphorylation sites. Such data can provide information about the function of the phosphorylation event as well as the nature of the kinase responsible for this phosphorylation. The two predominant techniques for phosphorylation site identification are i) Edman degradation and ii) MS analysis.

### Edman degradation

Edman degradation is still one of the most practical methods to determine phosphorylation sites in peptides [62-64]. This is because the technique is relatively simple, very sensitive, and can be applied to a large variety of peptides [28,31,65]. If enough radioactivity can be incorporated into the phosphoprotein of interest, sites can be determined at the sub-fmol level. In this approach, a ^32^P-labeled protein is digested with a protease and the resulting phosphopeptides are purified by reverse-phase HPLC or thin-layer chromatography (TLC). The isolated peptides then are cross-linked via their C-termini to an inert membrane and the radioactive membrane is subjected to several rounds of Edman cycles. The radioactivity is collected after each cleavage step and the released ^32^P is measured in a scintillation counter. This method positionally places the phosphoamino acid within the sequenced phosphopeptide. Of course, this is meaningful only if the sequence of the phosphopeptide is known. This method is not any more quantitative beyond 30 Edman cycles. In addition, the need for a large amount of starting material (more than pmol amounts of protein) and the length of time to completion has made this procedure prohibitive to high throughput studies of the phosphoproteome. However, MacDonald *et al*. have extended the usefulness of phosphorylation site characterization by Edman chemistry through the development of the cleaved radioactive peptide (CRP) program [66]. In CRP analysis, one requires only that the sequence of the protein to be known. Purification and sequencing of individual peptides is not required. Radiolabeled proteins are cleaved at predetermined residues by the action of a protease. The phosphopeptides are then separated by HPLC or TLC, cross-linked to the inert membrane, and carried through 25–30 Edman cycles. The sequence of the target protein is entered into the CRP program. This program predicts how many Edman cycles are required to cover 100% of the serines, threonines, and tyrosines from the site of cleavage. However, this methodology is still very low-throughput and require protein radiolabeling with ^32^P.

### MS analysis

Phosphorylation sites in peptides can also be analyzed by MS/MS. In this approach, the phosphopeptide is sequenced in the mass spectrometer and the site of phosphorylation is determined unambiguously. Precursor ion and neutral loss scanning are currently the methods of choice for sequencing phosphopeptides [58,67-70].

#### Precursor ion scanning

On fragmentation by collision-induced dissociation (CID) in a tandem mass spectrometer, phosphopeptides not only produce sequence-specific fragments but also fragments that are specific for phosphate groups. These phosphate-specific fragment ions serve as characteristic 'reporter ions' for phosphorylated peptides in precursor-ion scanning experiments by MS/MS [58]. In the negative ion mode phosphopeptides fragment produce marker ions at m/z 79 (PO_3_^-^) and 63 (PO_2_^-^). CID of phosphoserine- and phosphothreonine-containing peptides in the positive ion mode often yields a neutral loss of H_3_PO_4 _via β-elimination [67]. Peaks corresponding to this loss (98 from singly charged precursors, 49 from doubly charged precursors, etc.) are often the most abundant ions in the CID spectrum, although this is not invariably the case. Phosphotyrosine residues do not undergo β-elimination but do produce a characteristic immonium ion at m/z 216. Use of the characteristic immonium ion of phosphotyrosine was suggested as a possible marker for phosphotyrosine-containing peptides when using triple quadrupole instruments [71-73]. However, because of the possible number of other a, b, and y ions that give rise to signals at the same nominal mass 216 [71,74], high-resolution MS, such as a QSTAR Pulsar quadrupole time-of-flight tandem mass spectrometer equipped with a nanoelectrospray, are required to distinguish between the P-Tyr immonium ion and those generated by other a, b, and y ions [74,75]. Other methods use the negative-ion mode to determine the more comprehensive phosphopeptide composition of peptide mixtures by observing a fragment at m/z 79 Da for phosphopeptides that contain P-Ser and/or P-Thr [76,77]. A triple quadrupole mass spectrometer operating in negative ion mode is generally used. In this method, detection of the specific reporter ion identifies the corresponding precursor phosphopeptide ion by its mass to charge (m/z) value. In precursor ion scans, also known as parent ion scans, only those peptides that fragment to produce the chosen marker ion (m/z 79), produce peaks in the spectrum, screening out all other species. Subsequent sequencing of the corresponding phosphopeptide requires a change in polarity and re-buffering of the sample, which implies that this system is not readily amenable to liquid chromatography (LC)-MS-based approaches. Despite these shortcomings, the method is a powerful tool because of its high selectivity and sensitivity and its applicability for serine, threonine and tyrosine phosphorylated residues.

#### Neutral loss scanning

When peptides containing phosphoserine or phosphothreonine residues are subjected to CID, they commonly undergo a gas-phase β-elimination reaction, resulting in a neutral loss of phosphoric acid (H_3_PO_4_, -98 Da) or are dephosphorylated (HPO_3_, -80 Da). Phosphotyrosines, however, are generally more resistant to this loss. Ideally, precursor ion experiments can be performed on a triple quadruple mass spectrometer with an offset to detect phosphopeptide species that undergo such a loss. In the MS/MS spectrum, a spacing of 69 Da (owing to dehydroalanine) or 83 Da (owing to dehydroaminobutyric acid) indicates the exact location of phosphorylated serine and threonine residues, respectively. For instance, using this method, Wong *et al*. have identified three phosphorylated sites in the cytosolic domain of calnexin (Ser 534, Ser 544 and Ser 563) [78].

The drawbacks of this method are the incidence of false-positive signals as well as the fact that the charge state of the phosphopeptide has to be known in advance.

### Emerging trends

Although Edman degradation and MS/MS using CID are currently the methods of choice for sequencing phosphopeptides, alternatives have been demonstrated.

An alternative to these multi step procedures is data-dependent analysis in the course of a single LC-MS experiment [59,69,79]. Separation of tryptic peptides using LC is an excellent way to decrease the complexity of the sample. In a variation of this technique, a 2D chromatographic separation, first on a strong cation exchange and then on a C18 column, has been performed [80]. Precursor ion or neutral loss scanning is carried out until an ion is detected above a pre-established intensity threshold. The observed ion is then subjected to MS/MS to obtain its sequence. Coupling of nanoLC systems to a mass spectrometer is valuable because separation of peptides by the upfront LC step decreases the ion suppression effect observed in the case of phosphopeptides.

Tandem mass spectrometry using electron capture dissociation has been also applied to phosphorylation site mapping [81]. This non-random technique favors fragmentation of peptides along the peptide backbone; Ser(P) and Thr(P) residues retain their phosphates, greatly facilitating sequencing of the peptide and locating the site(s) of phosphorylation.

Electron capture dissociation (ECD) combined with Fourier transform ion cyclotron resonance (FTICR) MS has emerged as a powerful method for the sequencing of proteins and peptides as well as for the study of post-translational modifications [82]. It has also been successfully applied for the exact localization of phosphorylated residues in peptides [83].

Recently, Aebersold and coworkers [84] described an alternative general chemical strategy for the enrichment and subsequent mass spectrometric analysis of phosphopeptides. This improvement consists in the direct capture of phosphopeptides in a single-step reaction with a primary amine – containing solution polymer (in this case, a Generation-5 polyamidoamine dendrimer) rather than complicated chemical transformations. Selective reaction of phosphorylated peptides with the amine groups of the dendrimer produces phosphopeptide-polymer conjugates that are physically larger than unmodified peptides. Afterward, the phosphopeptides are released from the dendrimer under acidic conditions, and isolated from the dendrimer using the same membrane-based filtration device [84].

## Quantitative phosphoproteome analysis

Although defining the precise sites of phosphorylation yields important information that can be related to the biological function of phosphoproteins, the quantitative evaluation of the extent of phosphorylation at a given site or in relation to other phosphorylation sites, within the same protein, is critical for the interpretation in terms of biological significance. This is why quantitation of phosphorylation is particularly important. A given protein might be in more than one signaling pathway with different stimuli inducing overlapping patterns of phosphorylation. That means a given site might not be phosphorylated at all, phosphorylated in a minority of molecules or, in an extreme case, on all the molecules of that protein. When a population of molecules from unsynchronized cells is analyzed, this situation corresponds to detection of unphosphorylated, weakly phosphorylated or highly phosphorylated peptides containing the residue. Similarly, the ratio of phosphorylation of a protein on multiple residues might be crucial for its function.

Mass spectrometric approaches to quantitative phosphorylation generally use stable isotope dilution whereby two samples are differentially labeled with mass-encoded tags such that the samples can be mixed and analyzed simultaneously. Each phosphopeptides thus appears as two peaks in the mass spectrum, and the relative abundances of the peaks reflect the amount of the phosphopetide in each sample. This can be accomplished by metabolic labeling of proteins in cell culture [85,86] or subsequent chemical labeling of functional groups such as peptide N termini or C termini [87-89]. *In vitro *labeling [13] is used to quantitate proteins by labeling cysteine residue by a method designated isotope-coded affinity tagging (ICAT). A variation to this strategy is to introduce a biotin tag into phosphoserine and phosphothreonine residues by β-elimination and Michael addition reaction [90]. Other methods take advantage of this reaction for attaching different tags for a quantitative analysis of phosphorylation without any enrichment [90-92]. Several other methods that use chemical moieties to make peptides heavier have also been developed [93-95]. Other methods use the treatment with phosphatase in conjunction with isotopic labeling. In this approach the quantitative phosphorylation in individual samples is measured by dividing a sample in two, labeling each with the light/heavy forms of a mass tag and treating one sample with phosphatase before recombining the fractions [96,97]. An *in vivo *labeling has been used to label yeast proteins by growing them ^15^N-labeled media that labels all proteins without any further manipulation and thus to quantitate the extent of phosphorylation [85]. An alternative approach to label proteins *in vivo*, designated stable isotope labeling by amino acid in cell culture (SILAC), which uses amino acid containing a stable isotope, has been developed [98].

The advantage of using stable-isotope containing amino acids over media containing ^15^N is that it can be used in cases where the sequence is not known. It is also possible to couple *in vitro *and *in vivo *labeling methods, to quantitate the phosphorylation, using ^15^N-labeling and a method related to ICAT [99].

An alternative to labeling of phosphopeptides *in vivo *and *in vitro *is to run two samples separately, each with the same internal standard. The internal standard is a chemically synthesized heavier version of the phosphopeptide being analyzed, allowing absolute quantitation of phosphorylation levels [100,101]. Although this method is quite useful for small-scale analysis, it cannot be extended to high-throughput experiments because it involves prior knowledge of the phosphorylation site and production of a synthetic peptide of each site.

## Phosphoproteome validation

Some approaches have limitations in that phosphorylation sites may differ from what might take place within the cell. Thus, data validation is particularly important for phosphoproteins analysis. In this review, we provide two major methodologies which allow for high throughput validation of protein phosphorylation.

### Proteome chip technology

Ptacek *et al*. [102] have developed assays to measure substrate specificity in yeast directly at the level of kinase-substrate interaction. This assay is highly selective because a discrete set of substrates are recognized by each kinase. Nonetheless, phosphorylations that do not normally occur *in vivo *may be identified from this assay. These false positives may be due to either *in vitro *phosphorylation of proteins by kinases that normally reside in other cellular compartments and/or are expressed at different times, or through the absence of adaptor proteins that limit the kinase-substrate interactions. Combining their data with other information provides a useful method of detecting interactions likely to occur *in vivo*. Thus, proteome chip technology [103] offers many advantages for studying protein phosphorylation.

### Homogenous assays

#### AlphaScreen technology

The high throughput capability of the AlphaScreen technology can be used to detect and quantify phosphopeptides. Indeed, AlphaScreen is a bead-based non radioactive and homogeneous detection technology. An AlphaScreen signal is produced when the AlphaScreen acceptor and donor beads are brought into close proximity by molecular interaction occurring between binding partners captured on the beads mentioned above [104]. In the case of phosphoprotein/peptide detection, the Donor beads are functionalized using a specific antibody against the protein of interest or Streptavidin. The Acceptor beads are functionalized using either anti-PY antibodies or Lewis Metal ions. AlphaScreen allows quantifying various analyses by performing competition assays and extrapolating signals with a standard curve. We have developed a proteomic functional approach which combines both the sensitivity of mass spectrometry sequencing to identify phosphopeptides and the throughput of AlphaScreen for validation of identified targets (Caruso *et al*., in preparation). Thus, the sensitivity of mass spectrometry sequencing coupled to the detection and throughput capacity of the AlphaScreen technology allow identifying and validating the presence of phosphorylation sites in given proteins.

#### IMAP technology

Similarly, in this assays fluorescently labeled peptides are incubated with a kinase, the phosphorylated peptides bind to the IMAP reagent (a trivalent metal with high affinity for phosphates) causing an increase in the polarization of the fluorescence [105].

## Conclusion

Protein phosphorylation is one of the most challenging posttranslational modifications to study, mainly due to the low abundance and stoichiometry of this event. However, protein phosphorylation is critical for many cellular processes, which therefore rely on the efficient addition or removal of phosphate groups on specific amino acid residues (serine, threonine and tyrosine) of certain proteins. The phosphoproteome consists of the entire complement of phosphorylated proteins in cells, which is mapped or analysed not only for the identification of phosphorylation sites, but also for the quantitation of phosphorylation events in signal transduction pathways in a time-dependent manner. The analysis of the phosphoproteome relies on techniques such as radioactive labeling, mass spectrometry and Edman-sequencing, usually coupled to upstream enrichment steps, which are used to increase the amount of phosphorylated species in the monitoring step. Current techniques for the analysis of the phosphoproteome have been reviewed in this paper. Many advances have been made on the enrichment and detection of phosphoproteins, but these processes are still not straightforward for several reasons: the stoichiometry of phosphorylation is usually very low, the phosphorylated sites on proteins vary, signalling molecules are present at low abundance within cells, minor phosphorylation sites might be difficult to identify due to a very limited dynamic range of most analytical techniques to study phosphorylation, and precautions need to be taken to inhibit phosphatase activity during preparation and purification steps of cell lysates. The enrichment in phosphoprotein or phosphopeptide content, prior to the respective analysis, circumvents some of the challenges presented. Moreover, these approaches help to understand the intricate cellular networks and regulation of pathways, as well as identifying new proteins involved in these processes that might reveal potential therapeutic strategies.

## Competing interests

The author(s) declare that they have no competing interests.

## Authors' contributions

FD and EC wrote the review and approved the final manuscript.
